# Effectiveness of workplace interventions in rehabilitating musculoskeletal disorders and preventing its consequences among workers with physical and sedentary employment: systematic review protocol

**DOI:** 10.1186/s13643-019-1127-0

**Published:** 2019-08-27

**Authors:** Karina Glies Vincents Seeberg, Lars Louis Andersen, Elizabeth Bengtsen, Emil Sundstrup

**Affiliations:** 1 National Research Centre for the Working Environment (NRCWE), Lersø parkallé 105, DK-2100 Copenhagen Ø, Denmark; 2 Sport Sciences, Department of Health Science and Technology, Aalborg University, DK-9220 Aalborg, Denmark

**Keywords:** Musculoskeletal disease, Pain, Occupational, Workplace-based, Physical work, Sedentary work, Workers, Intervention

## Abstract

**Background:**

Musculoskeletal disorders (MSDs) are the leading cause of work disability and sickness absence worldwide. The prevalence of MSDs increases with age, consequently challenging sustainable employability among the growing ageing workforce. Knowledge of feasible and efficient workplace-based interventions to rehabilitate MSD or prevent its consequences is therefore warranted. This systematic review will evaluate the effectiveness of workplace-based interventions on MSDs and its consequences among adult workers with physical and sedentary work tasks, respectively.

**Methods:**

We will search the following bibliographic databases: PubMed (including the database ‘MEDLINE’) and Web of Science Core Collection (including the databases ‘Science Citation Index Expanded’, ‘Social Sciences Citation Index’ and ‘Arts & Humanities Citation Index’). Manual searches will also be performed. We will include randomised controlled trials (RCTs) and non-RCTs reported in English in which (1) participants are adult workers with MSD, (2) interventions are aiming at rehabilitating pain symptoms of MSD or preventing the consequences of MSD and (3) interventions are initiated and/or carried out at the workplace. The review will adhere to the ‘Preferred Reporting Items for Systematic reviews and Meta-Analyses’ (PRISMA) guidelines for reporting systematic reviews and the Institute for Work and Health (IWH) guidelines for workplace-based interventions. For the primary evaluation of the review, the quality assessment and evidence synthesis will conform to the IWH guidelines. Secondary evaluation will include a meta-analysis (unless the included studies do not allow this due to heterogeneity) and employ the risk of bias domains recommended by Cochrane along with the Grading of Recommendations, Assessment, Development and Evaluations (GRADE) approach on the studies with pain intensity as an outcome.

**Discussion:**

This systematic review will provide knowledge on effective workplace-based interventions among physical and sedentary workers with MSD. RCTs are considered the most powerful experimental design in clinical trials, but solely including these may be too restrictive to understand effective workplace-based interventions where randomised and carefully controlled trials (RCTs) are not always possible. In order to maximize practical relevance, the selection process will, therefore, include both RCTs and non-RCTs and the quality assessment and evidence synthesis will conform to IWH guidelines focusing on developing practical guidelines for stakeholders. The result of this work will form the basis for industry-specific evidence-based recommendations on effective workplace-based interventions for rehabilitation of MSDs and its consequences that will later be operationalised into concrete and user-friendly practical tools for workplaces.

**Systematic review registration:**

International Prospective Register of Systematic Reviews (PROSPERO) number CRD42018116752.

**Electronic supplementary material:**

The online version of this article (10.1186/s13643-019-1127-0) contains supplementary material, which is available to authorized users.

## Background

Musculoskeletal disorders (MSDs) continue to be a leading cause of disability worldwide [1]. MSD is defined by the World Health Organization as “health problems of the locomotor apparatus (i.e. of muscles, tendons, the skeleton, cartilage, ligaments, and nerves). MSD include all forms of ill-health ranging from light, transitory disorders to irreversible, disabling injuries” [2]. Thus, MSDs are a diverse group of conditions which affect the musculoskeletal system and are associated with pain and impaired physical function [3–6]. Within this diverse group of conditions, low back and neck pain are highly prevalent among workers and the leading causes of disability in high-income countries [7]. MSD has a multifactorial etiology consisting of a complex interaction between individual factors and physical and psychosocial work environmental factors [3–5, 8]. The prevalence of MSDs increases with age consequently challenging sustainable employability among the ageing workforce[7].

While some workers are able to work with their MSD, others may experience an imbalance between the demands at work and individual capacity, consequently increasing the risk of sickness absence and premature exit from the labour market [9–12]. Furthermore, psychosocial working conditions such as time pressure, low job decision latitude and lack of social support can augment the consequences of MSDs [2]. Knowledge of feasible and efficient intervention strategies to rehabilitate MSD or prevent its consequences is therefore warranted by workers, companies and societies.

### Physical work and MSD

MSD is a huge problem among the working population, and many people across occupation and industry will experience one or more episodes of MSDs during their lives. However, MSDs seem especially to be a problem among workers with hard physical work where pain may interfere with the ability to handle the physical work demands. Work-related musculoskeletal disorders are supposed to be causally linked to physical load resulting from occupational activities, and it is mainly caused by a mechanical overload [2]. Particularly among workers engaged in manual labour, physical work exposures—such as heavy lifting, pushing and pulling, bending and twisting of the back—play an important role in the development and retention of MSD. Previous studies have shown that workers exposed to physical demanding job tasks are at greatest risk of reporting low back pain [13].

### Sedentary work and MSD

Even though MSDs are more prevalent among physical workers, MSDs also occur in industries and occupations with more sedentary job tasks. In particular, office work or computer work has been associated with MSDs such as neck pain and trapezius myalgia [14–16]. Factors associated with MSD in office workers are workload, cognitive demands, hours worked at a computer, keyboard and mouse use, sustained awkward postures, psychological stress, psychosocial issues and design features of office workstations and buildings [2, 17]. Long-time sitting in a fixed posture is accompanied by long-lasting muscular activity which may lead to overload within the muscular structures [2, 17].

Because MSD is a global health issue in general and in particular in workers, employers, employees and other relevant stakeholders request knowledge of effective industry-specific interventions to prevent, manage or reduce MSD among workers in general (i.e. both among physical and sedentary workers).

### Description of the intervention

Risk factors for MSD along with effective interventions vary from industry to industry, and it is recommended that each industry and company face their specific and relevant risk factors in addition to the general knowledge about occupational risk factor. Industry-specific knowledge will, therefore, have the potential to better qualify the workplace’s choice of relevant, feasible and effective interventions to rehabilitate MSD and reduce its consequences. Although these overall factors associated with MSD in the working environment seem comparable, there may still be a large variation in types and doses of exposure between the job groups which may affect both the choice and outcome of a given intervention [18].

### Why it is important to do this review

Although there has been a major focus on rehabilitating MSD and preventing its consequences, MSD remains a significant problem among workers worldwide. Thus, workers, workplaces and other relevant stakeholders request knowledge on effective occupational industry-specific interventions to prevent and reduce MSD among workers.

Currently, there is no systematic review documenting and summarising the literature on the effect of workplace-based interventions specifically among physical and sedentary workers with different MSD conditions. Previous systematic reviews within this topic mainly focused on MSD in one body region or single joint complaints or among a specific job group (Additional file 1). Our systematic review will focus on the effect of all kinds of workplace-based interventions on all types of MSD in both physical and sedentary workers and will, therefore, embrace a broader area than other systematic reviews.

The aim of this systematic review is to identify, investigate and synthesise the evidence of the effect of workplace-based interventions on MSD and its consequences among workers in physical and sedentary employment.

The following review questions will be addressed by the review:

1. Which workplace interventions are effective in rehabilitating MSD and preventing its consequences among workers with physical jobs?
2. Which workplace interventions are effective in rehabilitating MSD and preventing its consequences among workers with sedentary jobs?

## Methods

The systematic review will follow the ‘Preferred Reporting Items for Systematic reviews and Meta-Analyses’ (PRISMA) guidelines for reporting systematic reviews and meta-analyses and the IWH guideline for workplace-based interventions. We will search PubMed and Web of Science Core Collection and first sort studies by title/abstract and then by full-text papers. Manual searches will also be performed. For the primary evaluation, both quality assessment and the evidence synthesis will conform to IWH guidelines. If the study allows a meta-analysis on the outcome pain intensity, a secondary evaluation will be performed where we employ the risk of bias domains recommended by Cochrane along with the GRADE approach on the studies with pain intensity as an outcome.

### Protocol and registration

The Cochrane standard including PRISMA statement may be too restrictive when investigating the effect of workplace-based interventions, where randomised and carefully controlled trials (RCTs) are not always possible. Therefore, the Cochrane standard may not be sufficiently comprehensive to employ for assessment of the quality and evidence of the included studies. As an alternative, the IWH group in Toronto has suggested more practice-oriented guidelines to prevent or reduce MSD focusing on developing practical recommendations to stakeholders [19–21]. The primary evaluation will, therefore, be based on the IWH guidelines for quality assessment and evidence synthesis (explained in detail below). We will follow the review steps according to both PRISMA guidelines [22] and the IWH guidelines for workplace-based interventions [21]. We will report the primary evaluation on the effect of workplace-based interventions on MSD in two distinct papers among workers in physical and sedentary employment with MSD. Our PRISMA flow diagram will remain the same for the two papers, except for the end stage where the included studies will be sorted by physical activity at work (i.e. physical or sedentary employment). For the two papers, the protocol will remain the same and we will, therefore, refer to the same PROSPERO registration number in both papers.

### Eligibility criteria

We will include studies on adult workers aged 18 years or older with any MSD (both specific and non-specific and including musculoskeletal pain). Any type of workplace-based intervention aiming at rehabilitating pain symptoms of MSD and/or preventing the consequences of MSD will be eligible. The intervention has to be initiated by the workplace and/or be performed at the workplace (i.e. workplace-based). Return-to-work interventions will not be included. Only studies with a comparison group will be included (i.e. no treatment, treatment as usual or another comparison treatment).

RCTs are generally considered the most powerful experimental design in clinical trials [23], but solely including these may be too restrictive to understand effective workplace interventions where randomised and carefully controlled trials are not always possible. Thus, both RCTs and non-RCTs (with a comparison group) will be eligible. Publication date will be imposed from 1998 to 2018. Only studies published in English will be eligible for inclusion.

### Types of outcome measures

Since MSDs are a diverse group of conditions, several different outcome measures have been employed in the literature. This heterogeneity may lead to challenges in relation to performing a meta-analysis in this systematic review since the outcomes will likely be too broad to be compared or pooled. Thus, we will not exclude potential relevant studies due to heterogeneity in outcomes, as long as they represent a sound measure for MSD. The outcomes presented below are therefore guidelines or inspirations for what we might find, not eligibility criteria.

### Primary outcome

For rehabilitation of pain symptom of MSD, the primary outcome will be the change in pain from baseline to follow-up. Pain is commonly measured in one of the following rating scales: numerical rating scale (NRS), verbal rating scale (VRS) and visual analog scale (VAS) [24].

### Other outcomes of interest

For the prevention of the consequences of MSD, we will — if possible — evaluate the interventional effect on other MSD-relevant outcomes such as work ability, work disability, sickness absence or productivity.

### Information sources and search

We will search the following bibliographic databases: PubMed (including the database ‘MEDLINE’) and Web of Science Core Collection (including the databases ‘Science Citation Index Expanded’, ‘Social Sciences Citation Index’ and ‘Arts & Humanities Citation Index’) from 1998 to 2018. The search strategy will have four main components/criteria which will be combined (1) musculoskeletal diseases/disorder AND (2) workers AND (3) intervention AND (4) date. Manual searches will also be performed by employing the ‘snowball’ method. Specifically, we will pursue references of paramount references within the field of MSD prevention at the workplace. In addition, relevant articles identified through personal knowledge and contacts will also be included in the review process [25].

The following search strategy will be applied in PubMed (Additional file 2) and in Web of Science Core Collection (Additional file 3).

### Study selection

Two independent reviewers will be involved in the study selection. Any discrepancies will be resolved by a consensus strategy. If consensus cannot be achieved, another author from the research group will act as arbitrator. EndNote X8 will be used to collect all potential items from PubMed and Web of Science Core Collection. The selected items will be exported to the review software programme Covidence. Two independent reviewers will start by screening title/abstracts using Covidence for potential studies meeting the inclusion criteria. The full-text papers of those potential studies will be assessed independently by two reviewers. The relevant studies, which adhere to the eligibility criteria, will be included in the systematic review. A PRISMA flow diagram will be generated to summarise the process of the study selection [22].

### Data collection process and data items

Data will be extracted on general characteristics of both RCT and non-RCT studies. Date of publication, sample size, industry/sector, body region of MSD, level of occupational physical activity (physical or sedentary employment) and study design will be extracted.

Unless the included studies do not allow a meta-analysis due to heterogeneity, data and risk of bias on the primary outcome (measured as pain intensity) will be calculated in Review Manager 5.3.

### Quality assessment in individual studies

Two reviewers will independently assess the quality of all full-text findings and use a consensus method if disagreements occur. For the primary evaluation, we will use the IWH quality assessment method. The IWH quality assessment score for each article will be based on a weighted sum score, and the categories will be determined by reviewer consensus with reference to other systematic reviews using this approach [21, 26]. The weighting values of the categories will range from 1 to 3. The rank score for each included study will be divided by the maximal weighted sum score and multiplied by 100. Finally, the studies will be divided into three groups depending on the ranking score: low quality (below 50%), medium quality (50–85%) and high quality (> 85%) [21, 27]. If the study allows a meta-analysis due to heterogeneity (on the outcome pain intensity), we will employ the risk of bias domains recommended by Cochrane along with the GRADE approach on the studies with pain intensity as an outcome [28–30]. The domains by Cochrane include sequence generation, allocation concealment, blinding, incomplete outcome data and selective outcome reporting. The risk of bias will be rated as low, high or unclear risk of bias [28].

### Summary measures and synthesis of results

We will use the IWH adapted ‘best evidence synthesis approach’ to clarify the evidence. Thus, conclusions and recommendations for the primary evaluation will be developed following the best evidence synthesis guidelines formulated by the IWH group. The approach considers the article’s quality, the quantity of articles evaluating the same intervention and finding consistency [21, 31]. Based on this, the level of evidence will be classified as ‘strong’, ‘moderate’, ‘limited’, ‘mixed’ or ‘insufficient’ based on the quality assessment of the included studies. A strong level of evidence will result in ‘recommendations’ for practice, and a moderate level of evidence will result in ‘practice recommendations’ or practices to be considered for workplace management of MSD [21, 31]. We will conduct overall recommendations from the systematic review for workers with MSD (overall and not body region-specific) among physical and sedentary workers.

If the included studies do not exclude a meta-analysis due to heterogeneity in, for example, pain measures, study design or intervention type, we will perform a secondary evaluation. We will use the Cochrane Q test [32] statistics for pain intensity to assess the statistical heterogeneity. The result will be evaluated as the *I*^2^ value. For the quantitative analysis of the primary outcome (i.e. pain), we will use a random effects model and plot the results of each trial as point estimates, odds ratios (OR) for dichotomous outcomes or mean differences (MD) and standard deviation (SD) for continuous outcomes.

If outcomes are deemed similar we will transform the point estimate to effect sizes or standardized mean differences [33]. For the effect sizes, we will use the generic inverse variance method as implemented in the Review Manager 5.3 to perform the meta-analysis. The GRADE approach will be followed to assess recommendations if a meta-analysis is possible [29, 30]. This secondary evaluation will also facilitate comparison with previous reviews using this approach.

### Additional analyses

To give more specific recommendations, we will stratify the included studies—if possible—on (1) occupational industry and (2) regional specific MSD. Regional specific MSD will include five sub-categories: (1) MSD in the arm, elbow and/or hand, (2) MSD in the neck and/or shoulder, (3) MSD in the back (including lower and/or upper back), (4) MSD in the head (including tension-type headache) and (5) MSD in the lower extremity (including hips, knees, legs and/or feet).

## Discussion

MSD is a huge public health problem, and even though there has been an increasing focus on obtaining knowledge on effective interventions for preventing and managing MSD, other systematic reviews mainly concern specific MSD diagnosis and/or body regional specific symptoms and often include workers from selected occupational industries or specific job groups [21, 34, 35]. We will investigate the effect of the interventions on workers with physical and sedentary work tasks, and—if possible—categorise the interventions by industry. This will allow us to provide recommendations and advices on how specific industries can manage and/or reduce MSD. The review will contribute to a subsequent process where relevant stakeholders and workplaces will be involved in the development of practical and user-friendly guidelines.

### Strength and limitations

Our systematic review will be performed and reported according to the PRISMA statement and the IWH guidelines. A highly sensitive search strategy will be used in order to identify as many relevant studies as possible and to reduce potential publication bias.

We will include both RCT and non-RCT studies in the systematic review. Some systematic reviews only include RCTs [36], but by only including these types of studies, we may exclude valuable information. RCTs are considered the most powerful experimental design in clinical trials [23], but solely including these may be too restrictive to understand effective workplace-based interventions where randomised and carefully controlled trials (RCTs) are not always possible. We are aware that we may downgrade the validity and strength of our systematic review and the risk of bias will become higher in the blinding and sequence generation domains when we include non-RCTs. Thus, we employ the IWH approach for the quality assessment and subsequent best evidence synthesis.

We expect high heterogeneity among the included studies, which may limit the relevance of performing a meta-analysis. However, if a meta-analysis is possible, we will perform a secondary evaluation where we employ the risk of bias domains recommended by Cochrane along with the GRADE approach on the studies with pain intensity as an outcome. We will only include studies restricted to English, which may exclude potential relevant studies reported in other languages.

The findings will not only provide guidelines for reducing MSD in workers but also identify research gaps for future workplace-based interventions for rehabilitating MSD.

### Dissemination

The primary evaluation of this systematic review will be reported in two papers: (1) effective workplace interventions among workers with MSD in physical employment and (2) effective workplace interventions among workers with MSD in sedentary employment. Secondary evaluation of this review (i.e. if a meta-analysis is possible) will be reported in two additional papers among workers with physical employment and sedentary employment, respectively.

## Additional files

Additional file 1: Overview of reviews. (DOCX 52 kb)

Additional file 2: Search strategy PubMed. (DOCX 20 kb)

Additional file 3: Search strategy Web of Science. (DOCX 15 kb)

## Data Availability

The data that support the findings of this review will be available from the corresponding author upon reasonable request.

## Abbreviations

GRADE: The Grading of Recommendations Assessment, Development and Evaluation
IWH: Institute of Work and Health, Toronto, Canada
MSDs: Musculoskeletal disorders
Non-RCT: Non-randomized controlled trial
NRS: Numerical rating scale
PRISMA: Preferred Reporting Items for Systematic reviews and Meta-Analyses
PROSPERO: International Prospective Register of Systematic Reviews
RCT: Randomised controlled trial
VAS: Visual analog scale
VRS: Verbal rating scale
