# Supplementary material for: Effectiveness of workplace interventions in rehabilitating musculoskeletal disorders and preventing its consequences among workers with physical and sedentary employment: systematic review protocol
Source: Syst Rev. 2019 Aug 27;8:219. doi: 10.1186/s13643-019-1127-0 (PMC6710868; doi:10.1186/s13643-019-1127-0)
Supplement: Supplementary file 1 — Overview of reviews. (DOCX 52 kb) [file 13643_2019_1127_MOESM1_ESM.docx]

| **Additional file 1: Overview of reviews** | | | | |
| --- | --- | --- | --- | --- |
| **Reference/guidelines** | **Year** | **Intervention** | **Aim** | **Result/outcome** |
| **Kennedy et al.(1)**  Systematic review of the role of occupational health and safety interventions in the prevention of upper extremity musculoskeletal symptoms, signs, disorder, injuries, claims and lost time.  **Institute for Work & Health (IWH) guidelines** | 2010 | Health and safety intervention   - **Exercise** - **Ergonomics training and exercise** - **Biofeedback** - **Cognitive behavioural training** - **Job stress management training** - **Workstation adjustment** - **Ergonomics training** - **Workstation adjustment and ergonomics training** - **Alternative keyboards** - **Rest breaks and exercise** - **Participatory ergonomics** | *“Do occupational health and safety interventions have an effect on upper extremity musculoskeletal symptoms, signs, disorder, injuries, claims and lost time?“*  87 studies met relevance criteria, 36 studies moved forward to the final review | Outcomes were measured as effect on the interventions.  Effect were measures as pain, symptoms, discomfort, incidents on MSD, time-loss claims, time-loss days, time-loss cost, complaints and sick-leaves.  *“Overall these studies provide mixed level of evidence for occupational health and safety interventions.”* |
| **Boocock et al.(2)**  Interventions for prevention and management of neck/upper extremity musculoskeletal conditions: a systematic review  **GATE**  **IWH** | 2007 | - **Work environment/workstation adjustments and visual display unit (VDU) workers** - **Workstation equipment and VDU workers** - **Ergonomic equipment and manufacturing workers.** - **Production systems/ organizational culture intervention** - **Modifier interventions;** - Exercise and neck/upper extremity conditions. - Exercise and fibromyalgia - Multiple modifier (including exercise) and neck/upper extremity conditions; - Multiple modifier (including exercise) and fibromyalgia; - Multiple modifier (excluding exercise) and neck/upper extremity conditions - Multiple modifier (excluding exercise) and fibromyalgia. | *“The aim was to evaluate the findings of primary, secondary and/ or tertiary interventions studies for neck/upper extremity condition.”*  31 intervention met the inclusion criteria | Outcomes were measured as pain intensity (VAS) , VADS, ergonomic rating scale, discomfort diary, modified and standard Nordic musculoskeletal questionnaire, clinical , medical and physical examination, JCI, WIRI, video-analysis, HAMA, SPADI, sick leave, PCS, BDI, PDI, FIQ, MPI-s, ASES-S, AIMS, QOLS, CPSS and EMG.  *“Overall these studies provide evidence to support the use of some mechanical and modifier interventions for prevention of upper extremity MSD and fibromyalgia.”* |
| **Martimo et al.(3)**  Effect of training and lifting equipment for preventing back  pain in lifting and handling: systematic review (meta –analysis)    **Preferred Reporting Items for Systematic Reviews and Meta-Analyses (PRISMA)** | 2008 | - Training interventions focused on **lifting technique**, with duration varying from a single session to training once a week for two years.   In three studies the training was supported by **follow up and feedback at the workplace**. Most studies used a professional instructor | The aim was *“To determine whether advice and training on*  *working techniques and lifting equipment prevent back*  *pain in jobs that involve heavy lifting”*  6 RCT and 5 cohort studies were included. | Outcomes were measured as back injury rate, pain and symptoms.  *“There is no evidence to support use of advice or training in working techniques with or without lifting equipment for preventing back pain or consequent disability. The findings challenge current widespread*  *practice of advising workers on correct lifting technique”* |
| **Sihawong et al.(4)**  Exercise therapy for office workers with  nonspecific neck pain: A systematic review  **PEDro-scale**  **PRISMA** | 2011 | - **Muscle strengthening exercise** - **Muscle endurance** - **Stretching exercise** - **Nonspecific exercise** | The aim of this study was to *“evaluate the effectiveness of various types of exercise for prevention and cure of nonspecific neck pain in office workers.”*  9 RCT were included | Outcomes were measured as  Discomfort, productivity, frequency of pain, severity of pain, sick-leave, recovery, disability, work ability and pressure pain threshold.  *“No exercise type was identified as being effective in the prevention of nonspecific neck pain in office workers.*  *Strong evidence was found for the effectiveness of muscle strengthening and endurance exercises in treating neck pain.*  *Moderate evidence supported the use of muscle endurance exercise in reducing disability attributed to neck pain.”* |
| **Verbeek et al.(5)**  Manual material handling advice and assistive devices for preventing and treating back pain in workers (Review)  **PRISMA** | 2011 | - **Manual material handling advice (MMH)** - **Assistive devices** | The aim was *“To determine the effectiveness of MMH advice and training and the provision of assistive devices in preventing and treating back pain.”*  9 RCT and 9 cohort studies | Outcomes were measured as incidence, duration, frequency of episodes or intensity or non-specific back pain and sick leave.  *“None of the included studies showed evidence of a preventive effect of training on back pain.”* |
| **Verhagen et al.(6)**  Exercise proves effective in a systematic review of work-related complaints of the arm, neck, or shoulder(CANS)  **PRISMA**  **Delphi list** | 2007 | - **Exercises** - **Behavioural therapy** - **Ergonomics** - **Massage** - **Group therapy vs. individual therapy** - **Manual therapy** - **Energised split** | The aim was to evaluate “whether conservative interventions have a significant impact on outcomes for work-related CANS.”  26 study were included 22 RCT 4 CCT | Outcome measures: pain, functional status (or quality of life), ability to work, health care consumption, and costs.  *“In conclusion, this review shows limited evidence for the efficacy of specific keyboards with an alternative force displacement or geometry only for patients with carpal tunnel syndrome.*  *There is limited evidence for the efficacy of exercises when compared to massage, adding breaks during computer work, massage as add-on treatment to manual therapy, and manual therapy as add-on treatment to exercises in patients with nonspecific work-related complaints.”* |
| **Verhagen et al.(7)**  Ergonomic and physiotherapeutic interventions for treating work-related complaints of the arm, neck or shoulder in adults- a Cochrane review  **PRISMA**  **Delphi list** | 2006 | - **Exercises** - **Ergonomics** - **Massage** - **Manual therapy/chiropractic treatment** - **Energised split** | The aim was to *“To determent the effects of conservative interventions for work-related CANS.”*  21 trials 3 CCT and 18 RCT | Outcome measures:  1. Pain (VAS), West Haven-Yale Multidimensional Pain Inventory (WHYMPI), ordinal scale)  2. Global status (e.g. overall improvement)  3. Functional status or quality of life (e.g. SF36, EQ5-D, Sickness Impact Profile, Health Assessment Questionnaire, Disabilities of the Arm, Shoulder and Hand Measurement Tool (DASH))  4. sickness absence, return to work, number of days off work  5. physicians' consultations, physiotherapy, ergonomic adjustments, intake of analgesics  6. Recurrence of injury  “There is limited evidence for the effectiveness of keyboards with an alternative force-dis-placement of the key or an alternative geometry and exercises compared to massage, brakes during computer work  Compared to no brakes; massage as an add-on treatment to manual therapy. And manual therapy as an ad-on treatment to exercise.” |
| **Van Hoof et al.(8)**  The efficacy of interventions for low back pain in nurses: A systematic  Review  **PRISMA**  **PROSPERO protocol** | 2018 | - **Manual handling training** - **Multidimensional interventions** - **Stretching exercises** - **Stress management** | The aim of this study was to *“investigate the efficacy of interventions for the prevention and treatment of low back pain in nurses.”*  14 studies were included, but 10 were excluded due to risk of bias. | Outcome measures:  Pain and/or disability, health care consumption.  From this systematic review it can be concluded that there is no strong evidence for any intervention in treating or preventing low back pain in nurses. |
| **Dick et al.(9)**  Workplacemanagement of upper limb disorders:  a systematic review  **SIGN protocol**  **PRISMA** | 2011 | - **A multi-disciplinary rehabilitation programme for workers** - **with non-specific musculoskeletal pain absent from work**   **for 90 days**   - **ergonomic training or ergonomic**   **interventions in the workplace**   - **stress management**   **training and an ergonomic intervention**   - **stress management interventions: progressive relaxation, applied**   **relaxation and Tai Chi**   - **massage as an add-on to physical therapy** - **a forearm support** - **modified keyboards** - **educational intervention** | The aim was *“To systematically review the evidence for workplace interventions in four common upper limb disorders.”*  28 studies were included but only 4 were used in the recommendation | Outcome measures:  Reduced pain, symptoms, complaints, sickness absence and sick leave  *“There was limited evidence that computer keyboards with altered force displacement characteristics or altered geometry were effective in reducing carpal tunnel syndrome symptoms.*  *This review found limited, but high quality, evidence that, for workers with non-specific arm pain who have been absent from work for at least 4 weeks, multi-disciplinary rehabilitation programs, including both physical and psychosocial approaches, may be beneficial. “* |
| **Skamagki et al.(10)**  A systematic review on workplace interventions to manage chronic musculoskeletal conditions  Publish 2008-2017  **PRISMA**  **SIGN tool** | 2018 | - **physical exercise** - **usual care** - **ergonomics** | The aim was *“to investigate whether there are effective*  *Workplace interventions that manage chronic musculoskeletal disorders.”*  The review included 12 studies | Outcome measures:  Pain intensity, pain symptom, functional status, pain duration, work ability, health status, work status, absenteeism, pain and sick leave.  *“There was some consistency in the results of the selected studies, suggesting*  *that workplace interventions such as high‐intensity strength exercises and/or*  *Integrated health care can decrease pain and symptoms for employees who experience long‐term musculoskeletal disorders. However, the current research is limited.”* |
| **Hoosain et al.(11)**  Workplace-Based Rehabilitation of Upper Limb Conditions:  A Systematic Review  **PRISMA**  **IWH** | 2019 | - **Job task adaptations** - **Job rotation** - **Alternate placement** - **Work environment/work station** - **Alternate ergonomic modifications** - **Stretching/exercise programs** - **Implementation of rest breaks at work** - **Work hardening** - **Negotiation with supervisors or managers** - **Splint application at work** - **Worker education and supervisor or manager education.** | *“The objective of this systematic review was to identify, collate and analyse the current available evidence on the*  *effectiveness of workplace-based rehabilitative interventions in workers with upper limb conditions on work performance,*  *pain, absenteeism, productivity and other outcomes”*  The review included 17 studies | Outcome measures:  Pain, headache, sick leave, upper limb postures, hand activity, symptoms, functional status scale, adjustment, use of brakes, works stress, ergonomic exposure, medical care, ergonomic risk assessment, productivity loss at work, pain intensity, sickness absence, tenderness, muscle strength, upper limb function, endurance errors in exercise execution, work ability, mental health, social climate, vitality, SPADI, FCE, return to work, recommended accommodations, complaints and. disability  *“There is substantial evidence for workplace exercise programs; other workplace-based interventions require further high quality research.”* |

# References

1. Kennedy CA, Amick BC, 3rd, Dennerlein JT, Brewer S, Catli S, Williams R, et al. Systematic review of the role of occupational health and safety interventions in the prevention of upper extremity musculoskeletal symptoms, signs, disorders, injuries, claims and lost time. Journal of occupational rehabilitation. 2010;20(2):127-62.

2. Boocock MG, McNair PJ, Larmer PJ, Armstrong B, Collier J, Simmonds M, et al. Interventions for the prevention and management of neck/upper extremity musculoskeletal conditions: a systematic review. Occupational and environmental medicine. 2007;64(5):291-303.

3. Martimo KP, Verbeek J, Karppinen J, Furlan AD, Takala EP, Kuijer PP, et al. Effect of training and lifting equipment for preventing back pain in lifting and handling: systematic review. BMJ (Clinical research ed). 2008;336(7641):429-31.

4. Sihawong R, Janwantanakul P, Sitthipornvorakul E, Pensri P. Exercise therapy for office workers with nonspecific neck pain: a systematic review. Journal of manipulative and physiological therapeutics. 2011;34(1):62-71.

5. Verbeek JH, Martimo KP, Karppinen J, Kuijer PP, Viikari-Juntura E, Takala EP. Manual material handling advice and assistive devices for preventing and treating back pain in workers. The Cochrane database of systematic reviews. 2011(6):Cd005958.

6. Verhagen AP, Karels C, Bierma-Zeinstra SM, Feleus A, Dahaghin S, Burdorf A, et al. Exercise proves effective in a systematic review of work-related complaints of the arm, neck, or shoulder. Journal of clinical epidemiology. 2007;60(2):110-7.

7. Verhagen AP, Karels C, Bierma-Zeinstra SM, Burdorf L, Feleus A, Dahaghin S, et al. Ergonomic and physiotherapeutic interventions for treating work-related complaints of the arm, neck or shoulder in adults. The Cochrane database of systematic reviews. 2006(3):Cd003471.

8. Van Hoof W, O'Sullivan K, O'Keeffe M, Verschueren S, O'Sullivan P, Dankaerts W. The efficacy of interventions for low back pain in nurses: A systematic review. International journal of nursing studies. 2018;77:222-31.

9. Dick FD, Graveling RA, Munro W, Walker-Bone K. Workplace management of upper limb disorders: a systematic review. Occupational medicine (Oxford, England). 2011;61(1):19-25.

10. Skamagki G, King A, Duncan M, Wahlin C. A systematic review on workplace interventions to manage chronic musculoskeletal conditions. Physiotherapy research international : the journal for researchers and clinicians in physical therapy. 2018:e1738.

11. Hoosain M, de Klerk S, Burger M. Workplace-Based Rehabilitation of Upper Limb Conditions: A Systematic Review. Journal of occupational rehabilitation. 2019;29(1):175-93.
