# Supplementary material for: Effectiveness of workplace interventions in rehabilitating musculoskeletal disorders and preventing its consequences among workers with physical and sedentary employment: systematic review protocol
Source: Syst Rev. 2019 Aug 27;8:219. doi: 10.1186/s13643-019-1127-0 (PMC6710868; doi:10.1186/s13643-019-1127-0)
Supplement: Supplementary file 2 — Search strategy PubMed. (DOCX 20 kb) [file 13643_2019_1127_MOESM2_ESM.docx]

| **Additional file 2: Search strategy PubMed** | |
| --- | --- |
| **Domains** | **Search string** |
| Condition  Musculoskeletal disorders/diseases | (((("lower limb"[Title/Abstract] OR "upper limb"[Title/Abstract] OR "Tension-Type Headache"[MeSH Terms]) OR ((((((((((((((((((((((((((((((((((((("musculoskeletal health"[Text Word]) OR "musculoskeletal diseases"[MeSH Terms]) OR "musculoskeletal diseases") OR "musculoskeletal disorder") OR "low back pain"[MeSH Terms]) OR "low back pain"[Text Word]) OR "upper extremity"[MeSH Terms]) OR "upper extremity"[Text Word]) OR "Musculoskeletal Pain"[Text Word]) OR "chronic nonmalignant pain"[Text Word]) OR ((("Chronic pain"[Text Word] AND nonmalignant[Text Word])))) OR Neck-shoulder pain[Text Word]) OR Neck/shoulder pain[Text Word]) OR neck/shoulder[Text Word]) OR "muscle pain"[Text Word]) OR "Neck pain"[Title/Abstract]) OR "Shoulder pain"[Title/Abstract]) OR "Knee pain"[Title/Abstract]) OR "Foot pain"[Title/Abstract]) OR "elbow pain"[Title/Abstract]) OR "Hip pain"[Title/Abstract]) OR "Hand pain"[Title/Abstract]) OR "back pain"[Title]) OR myalgia[MeSH Terms])) OR "back pain"[Title/Abstract]))) OR ((((((("musculoskeletal health") OR musculoskeletal) OR "musculoskeletal disorder"[Text Word]) OR "musculoskeletal diseases"[Text Word]) OR "low back pain") OR "upper extremity") OR "Musculoskeletal Pain")))))))))) |
| AND | |
| Workers and work | (computer user[Text Word]) OR ((((((((((((((((((((((((((((((((((((((((((((((((((((((((((("occupational groups"[Text Word]) OR "occupational groups"[MeSH Terms]) OR Industry[MeSH Terms]) OR Employment[MeSH Terms]) OR employer[Text Word]) OR factory[Text Word]) OR factory[MeSH Terms]) OR factories[Text Word]) OR factories[MeSH Terms]) OR Office[Journal]) OR Office[Text Word]) OR company[Text Word]) OR company[MeSH Terms]) OR companies[Text Word]) OR onsite[Text Word]) OR employee[Text Word]) OR worker[Text Word]) OR manpower[Subheading]) OR manpower[Text Word]) OR workers[Text Word]) OR workers[MeSH Terms]) OR "company physician"[MeSH Terms]) OR company physician[MeSH Terms]) OR physician practice management company[MeSH Terms]) OR "physician practice management company"[MeSH Terms])) OR construction industry[MeSH Terms]) OR "construction industry"[MeSH Terms]) OR building industry[MeSH Terms]) OR "building industry"[MeSH Terms]) OR health care industry[MeSH Terms]) OR "health care industry"[MeSH Terms]) OR healthcare industry[MeSH Terms]) OR "healthcare industry"[MeSH Terms]) OR heavy industry[MeSH Terms]) OR "heavy industry"[MeSH Terms]) OR industry[MeSH Terms]) OR firm[Text Word]) OR personnel[MeSH Terms]) OR employee*[Text Word]) OR employment[MeSH Terms]) OR labourer*[Title/Abstract]) OR laborer*[Title/Abstract] OR occupations[MeSH Terms]) OR occupation*[Text Word]) OR worker*[Title/Abstract]) OR workforce[Title/Abstract] OR employees[Text Word])))))))))))))))) |
| AND | |
| Intervention | (((((((((((((((((((((((((((((((((((((((((((((((((((((((((((((((((((((((((((((((((ergonomic program[Title/Abstract]) OR "ergonomic programme"[Title/Abstract]) OR "exercise training"[Title]) OR "flexible work"[Title/Abstract]) OR "flexible worker"[Title/Abstract]) OR "functional training"[Title/Abstract]) OR "functional ability"[Title/Abstract])) OR "functional abilities"[Title]) OR "functional capacity"[Title/Abstract]) OR "graded activity"[Title/Abstract]) OR "graded work"[Title])) OR graduated hours[Title/Abstract]) OR health care provider training[Title/Abstract]) OR health care provider training[Text Word]) OR healthcare provider training[Title/Abstract]) OR "healthcare provider training"[Title/Abstract])) OR "healthcare provider training"[Title/Abstract]) OR "human resource training"[Title/Abstract]) OR job accommodation[Title/Abstract]) OR job adaption[Title/Abstract]) OR job person[Title/Abstract]) OR light duties[Title/Abstract]) OR Light duty[Title/Abstract]) OR light work[Title/Abstract]) OR "light work"[Title/Abstract]) OR modified duties[Title/Abstract]) OR modified duty[Title/Abstract]) OR modified work[Title/Abstract]) OR "motivational interviewing"[Title/Abstract]) OR multidisciplinary intervention[Title]) OR "occupational health"[MeSH Terms]) OR "guidelines as topic"[MeSH Terms]) OR "occupational health guidelines"[Title/Abstract])) OR "occupational intervention"[Title/Abstract]) OR occupational management[Title]) OR "occupational rehabilitation"[Title/Abstract]) OR "pain reduction"[Title/Abstract]) OR "participatory ergonomics"[Title/Abstract]) OR participatory ergonomics[Text Word]) OR psychotherapy[Title/Abstract]) OR psychotherapy[MeSH Terms]) OR reasonable accommodation[Title/Abstract]) OR reasonable adjustment[Title]) OR "secondary prevention"[Title/Abstract]) OR self-management training[Title/Abstract]) OR service coordination[Title/Abstract]) OR "stress management"[Title/Abstract]) OR "structured rehabilitation program"[Title/Abstract])) OR suitable duties[Title/Abstract]) OR supervisor[Title/Abstract]) OR supportive colleagues[Title/Abstract]) OR supportive manager[Title/Abstract]) OR vocational assessment[Title/Abstract]) OR "vocational rehabilitation"[Title/Abstract]) OR work adjustment[Title]) OR work conditioning[Title/Abstract]) OR work-directed interventions[Title/Abstract]) OR "work-directed interventions"[Title/Abstract]) OR work disability management[Title/Abstract]) OR work hardening[Title/Abstract]) OR work modification[Title/Abstract]) OR "work program"[Title/Abstract]) OR work interventions[Title/Abstract]) OR work intervention[Title/Abstract]) OR "working intervention"[Title/Abstract]) OR working interventions[Title/Abstract]) OR work trial[Title/Abstract] OR working trials[Title/Abstract] OR work trials[Title/Abstract] OR "working trial"[Title/Abstract] OR (((((((((((((((((((((((((((("work-place based intervention"[Text Word]) OR "work-place based intervention"[MeSH Terms]) OR "workplace based intervention"[Text Word]) OR "workplace-based intervention"[Text Word]) OR "combined modality therapy"[Title/Abstract]) OR "combined modality therapy"[Text Word]) OR "combined modality therapy"[MeSH Terms]) OR "cognitive behavioral interventions"[Title/Abstract]) OR "cognitive behavioral interventions"[Text Word]) OR "cognitive behavioral principles"[Title/Abstract]) OR "cognitive therapy"[MeSH Terms]) OR "compensation management"[Title/Abstract]) OR "coordinated program"[Title/Abstract]) OR "disability management"[Title]) OR "disclosure management"[Title/Abstract]) OR "early intervention"[Title]) OR "employee assistance"[Title/Abstract]) OR "employer accommodation"[Title/Abstract]) OR "employer contact"[Title/Abstract]) OR "ergonomic approaches"[Title/Abstract]) OR "ergonomic intervention"[Title/Abstract])))))) OR (((((((((((((((((((((((("workplace intervention"[Title/Abstract]) OR "workplace interventions"[Title/Abstract]) OR workplace linked[Title/Abstract]) OR workplace modification[Title/Abstract]) OR "behavior therapy"[Title/Abstract]) OR behaviour therapy[Title/Abstract]) OR behavior therapy[MeSH Terms]) OR attendance management[Text Word]) OR alternative work[Text Word]) OR absence management[Text Word]) OR Rest breaks[Text Word]) OR "prevention strategies"[Text Word]) OR "physical therapy"[Text Word]) OR "multi-component patient handling"[Text Word]) OR "new chair"[Text Word])) OR arm supports[Title/Abstract]) OR alternative pointing devices[Text Word]) OR "alternative keyboards"[Text Word])))) OR (((((((((((((((((((((((((("ergonomics training"[Text Word]) OR "workstation adjustment"[Text Word]) OR "job stress management training"[Title/Abstract]) OR "job stress management training"[Text Word]) OR cognitive behavioral training[Title/Abstract]) OR "biofeedback training"[Text Word])) OR "cognitive behavioral training"[Text Word]) OR job stress management training[Text Word]) OR "Physical Therapy Modalities"[MeSH Terms]) OR "complementary Therapies"[MeSH Terms]) OR Exercise Therapy[MeSH Terms]) OR Exercise[MeSH Terms]) OR "Modified work"[Text Word]) OR Modified job[Text Word]) OR occupational therapy[MeSH Terms]) OR "occupational Therapy"[Text Word]) OR ergonomics[MeSH Terms]) OR Participatory[Text Word]) OR Participatories[Text Word]) OR work site[Title]) OR worksite*[Title/Abstract]) OR work based[Title/Abstract]) OR workplace[MeSH Terms]) OR workplace[Text Word]) OR training[Text Word]) OR exercise[Text Word])))))))) OR training[Title] OR "self-management programme"[Title/Abstract]))) |
| AND | |
| Publication date | ("1998/01/01"[Date - Publication] : "2018/10/01"[Date - Publication]) |
| NOT | |
| Types of items | ((((((((letter [pt] OR newspaper article [pt]))) OR (((systematic review [ti] OR meta-analysis [pt] OR meta-analysis [ti] OR systematic literature review [ti] OR this systematic review [tw] OR pooling project [tw] OR (systematic review [tiab] AND review [pt]) OR meta synthesis [ti] OR meta-analy*[ti] OR integrative review [tw] OR integrative research review [tw] OR rapid review [tw] OR umbrella review [tw] OR consensus development conference [pt] OR practice guideline [pt] OR drug class reviews [ti] OR cochrane database syst rev [ta] OR acp journal club [ta] OR health technol assess [ta] OR evid rep technol assess summ [ta] OR jbi database system rev implement rep [ta])))) OR review [pt]) OR ((review [tiab] OR reviews [tiab]))) OR meta-analy* [tw]) OR bibliography [tiab]) OR bibliographies [tiab] |
