# Supplementary material for: Effectiveness of workplace interventions in rehabilitating musculoskeletal disorders and preventing its consequences among workers with physical and sedentary employment: systematic review protocol
Source: Syst Rev. 2019 Aug 27;8:219. doi: 10.1186/s13643-019-1127-0 (PMC6710868; doi:10.1186/s13643-019-1127-0)
Supplement: Supplementary file 3 — Search strategy Web of Science. (DOCX 15 kb) [file 13643_2019_1127_MOESM3_ESM.docx]

| **Additional file 3: Search strategy Web Of Science** | |
| --- | --- |
| **Domains** | **Search string** |
| Musculoskeletal disorder/diseases | TS=("musculoskeletal health” OR "musculoskeletal diseases" OR "musculoskeletal disorder" OR "low back pain” OR "upper extremity" OR "Musculoskeletal Pain" OR "chronic nonmalignant pain" OR “Neck-shoulder pain” OR “Neck/shoulder pain” OR “muscle pain” OR "Neck pain" OR "Shoulder pain" OR "Knee pain" OR "Foot pain" OR "elbow pain” OR "Hip pain" OR "Hand pain" OR "back pain" OR musculoskeletal OR “upper limb” OR “lower limb” OR “tension-type headache”) |
| AND | |
| Workers | TS=("occupational groups" OR “occupational group” OR employment OR employer OR factory OR factories OR Office OR company OR companies OR onsite OR manpower OR "company physician" OR "physician practice management company" OR “construction industry” OR "building industry" OR "health care industry" OR “healthcare industry” OR "heavy industry" OR firm OR personnel OR employee* OR employment OR labourer* OR occupation* OR worker* OR workforce OR industry OR job OR workplace OR “computer user” OR “computer users”) |
| AND | |
| Intervention | TS=(intervention OR ergonomic OR exercise OR training OR workplace OR "light duty" OR ((motivational) NEAR/2 (interviewing)) OR ((guidelines) NEAR/2 (topic)) OR ((pain) NEAR/2 (reduction)) OR psychotherapy OR ((reasonable) NEAR/2 (accommodation)) OR ((reasonable) NEAR/2 (adjustment)) OR ((service) NEAR/2 (coordination)) OR ((stress) NEAR/2 (management)) OR ((structured) NEAR/2 (rehabilitation) NEAR/2 (program)) OR supervisor OR ((supportive) NEAR/2 (colleagues)) OR ((supportive) NEAR/2 (manager)) OR ((vocational) NEAR/2 (rehabilitation)) OR ((cognitive) NEAR/2 (behavioral) NEAR/2 (principles)) OR ((cognitive) NEAR/2 (behavioural) NEAR/2 (principles)) OR ((coordinated) NEAR/2 (program)) OR ((disability) NEAR/2 (management)) OR ((disclosure) NEAR/2 (management)) OR ((employee) NEAR/2 (assistance)) OR ((employer) NEAR/2 (accommodation)) OR ((employer) NEAR/2 (contact)) OR ((ergonomic) NEAR/2 (approaches)) OR ((attendance) NEAR/2 (management)) OR ((absence) NEAR/2 (management)) OR ((Rest) NEAR/2 (breaks)) OR prevention OR multi-component patient handling OR "new chair" OR ((alternative) NEAR/2 (pointing devices)) OR ((alternative) NEAR/2 (keyboards)) OR ((workstation) NEAR/2 (adjustment)) OR Participatory OR worksite* OR ((self-management) NEAR/2 (programme))) |
| **Filters in web of science** | |
| Date | 1998-2018 |
| Language | English, Danish |
| Document Types | Article |
